# Breast conservation, mastectomy and axillary surgery in New South Wales women in 1992 and 1995

**DOI:** 10.1054/bjoc.2001.1932

**Published:** 2001-09

**Authors:** A Kricker, J Haskill, B K Armstrong

**Affiliations:** National Breast Cancer Centre, NSW Cancer Council, Sydney, Australia and; Cancer Research and Registers Division, NSW Cancer Council, Sydney, Australia

**Keywords:** breast cancer, breast conservation therapy, mastectomy, axillary surgery

## Abstract

To measure the increase in uptake of BCT in NSW and its determinants, we examined Cancer Registry records of 2020 women with breast cancer in 1992 and 2883 in 1995 linked to records of their surgical treatment in the NSW Inpatient Statistics' Collection. In parallel, we examined trends and determinants in axillary surgery for breast cancer. Breast conservation increased from 39% of breast cancer in 1992 to 45% in 1995, mainly in women with the smallest cancers. In 1995, mastectomy was still most common in women with larger cancers (OR for breast cancers 3+ cm relative to <1 cm = 5.6, 95% CI 2.9–10.7) and cancers that had spread beyond the breast (OR = 2.0, 95% CI 1.4–2.7 relative to localized to the breast). Urban women had fewer mastectomies than rural women. Axillary surgery, common in 1992 (78%) and 1995 (82%), fell steeply with increasing age and more often accompanied mastectomy (93% in 1995) than BCT (67% in 1995). In 1995 the odds for axillary surgery were some two-fold or more higher for all cancers 1 cm or more in diameter compared with those <1.0 cm and highest for 2.0–2.9 cm cancers (OR = 3.3 95% CI 1.7–6.7 relative to <1.0 cm). Regional spread of the cancer at diagnosis was not a strong predictor. In the absence of collection of treatment data by cancer registries, linkage of cancer registry records with hospital inpatient data is an effective alternative for monitoring breast cancer treatment trends. © 2001 Cancer Research Campaign http://www.bjcancer.com


*British Journal of Cancer* (2001) **85**(5), 668–673
© 2001 Cancer Research Campaign
doi: 10.1054/ bjoc.2000.1932, available online at http://www.idealibrary.com on http://www.bjcancer.com

### Breast conservation, mastectomy and axillary surgery in New South Wales women in 1992 and 1995

**A Kricker[1], J Haskill[1] and BK Armstrong[2]**

[1]National Breast Cancer Centre, NSW Cancer Council, Sydney, Australia and [2]Cancer Research and Registers Division, NSW Cancer Council, Sydney, Australia

**Summary** To measure the increase in uptake of BCT in NSW and its determinants, we examined Cancer Registry records of 2020 women with breast cancer in 1992 and 2883 in 1995 linked to records of their surgical treatment in the NSW Inpatient Statistics' Collection. In parallel, we examined trends and determinants in axillary surgery for breast cancer. Breast conservation increased from 39% of breast cancer in 1992 to 45% in 1995, mainly in women with the smallest cancers. In 1995, mastectomy was still most common in women with larger cancers (OR for breast cancers 3+ cm relative to <1 cm = 5.6, 95% CI 2.9–10.7) and cancers that had spread beyond the breast (OR = 2.0, 95% CI 1.4–2.7 relative to localized to the breast). Urban women had fewer mastectomies than rural women. Axillary surgery, common in 1992 (78%) and 1995 (82%), fell steeply with increasing age and more often accompanied mastectomy (93% in 1995) than BCT (67% in 1995). In 1995 the odds for axillary surgery were some two-fold or more higher for all cancers 1 cm or more in diameter compared with those <1.0 cm and highest for 2.0–2.9 cm cancers (OR = 3.3 95% CI 1.7–6.7 relative to <1.0 cm). Regional spread of the cancer at diagnosis was not a strong predictor. In the absence of collection of treatment data by cancer registries, linkage of cancer registry records with hospital inpatient data is an effective alternative for monitoring breast cancer treatment trends. © 2001 Cancer Research Campaign http://www.bjcancer.com

**Keywords**: breast cancer; breast conservation therapy; mastectomy; axillary surgery

Breast-conserving therapy (BCT) is accepted as effective as mastectomy in treating early breast cancer. In June 1990, a Consensus Development Conference of the US National Institutes of Health concluded that breast conservation was appropriate for most women with Stage I and II breast cancer and was preferable to total mastectomy because it provided equivalent survival while preserving the breast (National Institute of Health, 1990). This position has been taken in other national and professional guidelines (National Health & Medical Research Council, 1995; Haward et al, 1999).

Changing established patterns of practice in areas such as clinical management of breast cancer usually takes time (Haward et al, 1999). Nonetheless, the release of information about treatment from clinical trials of the 1980s and the consensus statements that followed are widely credited with an increased uptake of BCT in the mid-to late 1980s and early 1990s in Europe and USA. Population-based reports on trends in breast cancer treatment, however, have been few in number (de Koning et al, 1994), mainly from the US SEER (Nattinger et al, 1996; Nattinger et al, 2000) and Yorkshire registries (Haward et al, 1999). Nationwide treatment data were available in the Netherlands, but not breast cancer registrations (de Koning et al, 1994).

Examination of population-based trends in breast cancer treatment depends on population-based cancer registration and recording of treatment. A minority of registries, however, such as the SEER (Lazovich et al, 1997; Nattinger et al, 2000) and some UK registries (Haward et al, 1999) collect treatment data, usually by sending trained abstractors to local hospitals. In its absence, and hoc or continuing linkage of routinely collected hospital inpatient records with cancer registry records is a potentially workable alternative to meet needs for population-based monitoring (Hobbs and McCall, 1970; Baldwin, 1972; Kahn et al, 1996; McGeechan et al, 1998).

*Received 28 November 2000*
*Revised 9 May 2001*
*Accepted 9 May 2001*

*Correspondence to:* A Kricker,

We used linked cancer registry records and inpatient records of surgical management in New South Wales (NSW), the most populous Australian state, to examine changes in treatment for breast cancer in Australia between 1992 and 1995. Specifically, we sought to use these records to examine whether management practice had changed to greater uptake of breast conservation surgery and, if so, to study demographic and clinical correlates of the changes.

### SUBJECTS AND METHODS

All cases of invasive breast cancer in women registered with breast cancer in the population-based NSW Central Cancer Registry in 1992 and 1995 were eligible for this study. The NSW Department of Health had linked Cancer Registry records of women diagnosed with breast cancer in 1992 and 1995 with their NSW Inpatient Statistics Collection (ISC) records for separations from any public or private hospital up to 1 year before and 18 months after their diagnosis. Linkage was probabilistic, using Automatch software, and matched records by date of birth, hospital code, patient's medical record number, country of birth and address of residence; the women's names were not used.

ICD9–CM procedure codes in the ISC records were used to classify each woman into one of five groups: mastectomy (ICD9–CM 85.41–85.48), breast conserving therapy (85.20–85.23), breast conserving therapy and mastectomy, diagnostic breast procedures only (85.0, 85.11, 85.12, 85.19), and no breast procedure. Analyses of therapeutic surgical procedures used two categories, women who had mastectomy (with or without BCT) and those who had

BJOC 01-1932 668-673 20/8/01 3:10 pm Page 669

BCT only. Separate analyses examined use of axillary surgery. Women were considered to have had axillary surgery when there was a code for axillary surgery (ICD9–CM 40.22, 40.23, 40.29, 40.3, 40.51) or for surgery to the lymph nodes with mastectomy (85.43–85.48). Hospital admissions for surgery were counted up to 12 months after diagnosis.

Age was analysed in broad age groups, country of birth in three categories (Australia, UK and Europe, other countries) and residence at the admission closest to the date of diagnosis as urban or rural according to a standard coding scheme. Area of residence was used to categorize the women into one of five socio-economic (SES) groups based on an index of relative socio-economic disadvantage for local government areas (LGAs). The index, derived by the Australian Bureau of Statistics from the 1991 and 1996 Australian Censuses, was used to rank LGAs and divide them into five SES groups of approximately equal population size. Spread of cancer at diagnosis was classified as localized to the breast, invading adjacent tissue or regional lymph nodes or distant metastases. Breast cancer size was available for women 40–69 years for the whole of 1992 and for women of all ages in 6 months of 1995 (Kricker et al, 1999) and was grouped into four categories (0.1–0.9 cm, 1.0–1.9 cm, 2.0–2.9 cm and 3.0+ cm).

Hospitals were classified as urban or rural and private or public, and also grouped into categories of 'surgical volume' by use of their numbers of therapeutic surgical procedures for breast cancer in 1992 and 1995. The categories used were low (1–10 procedures), intermediate (11–20 procedures) and high (21 + procedures).

Predictors of mastectomy and axillary surgery in 1992 and 1995 were examined separately in logistic regression models, initially within subsets of variables relating to the woman (age, place of residence, SES of area of residence and country of birth), the cancer (histopathological type, extent of disease, and cancer size in women 40–69 years of age) and the hospital (volume of surgery for breast cancer, urban or rural location and public or private status). Variables that were independent predictors of the procedure of interest within each subset in either year were examined together in separate models for 1992 and 1995.

### RESULTS

Most of the Cancer Registry records of women with invasive breast cancer were successfully linked to ISC records in 1992 (93%) and 1995 (96%). All analyses were based on the linked records.

### Breast surgery

Hospital admissions for BCT or mastectomy were recorded for 2020 women in 1992 and 2883 in 1995. A mastectomy was done within 12 months of diagnosis in 61% of women in 1992 and 55% in 1995. Breast conservation as the only surgical procedure increased from 39% in 1992 to 45% in 1995.

Urban women were less likely to have mastectomy than rural women (Table 1) and this gap did not change between 1992 and 1995 (Tables 1 and 4).

In 1992, women 80 years of age and older (OR 0.6, 95% CI 0.4–0.8) and 40–49 years (OR 0.8, 95% CI 0.6–1.0) were less likely to have mastectomy than women 50–59 years (baseline age group; *P* for heterogeneity with age 0.02; Table 2). Women 25–39 and 60–79 years had similar odds of mastectomy to women 50–59 years. In 1995 the odds for mastectomy varied less and were not significantly heterogeneous by age ($P = 0.3$, Table 2).

Analyses of the relationship between breast cancer size and mastectomy were restricted to women 40–69 years of age for whom cancer size was known (Table 3). Mastectomy increased with increasing cancer size in urban women in both 1992 and 1995. The pattern in rural women was less clear, except that the greatest use of mastectomy was in women with the largest (3+ cm) cancers. The percentage of women with 3+ cm cancers who had mastectomy changed little between 1992 and 1995. On the other hand, the percentage who had mastectomy for <1 cm cancers fell from 50% to 36% in urban women and 64% to 47% in rural women. Most of the shift from mastectomy to BCT in this period occurred in these smallest cancers.

The odds of mastectomy were two-fold higher in the presence of regional spread of cancer at diagnosis than with localized cancer (Table 4). It was not increased in those with distant spread.

**Table 1** Numbers of urban and rural women who had surgery and percentages of urban and rural women who had mastectomy or breast conservation and axillary surgery for breast cancer in 1992 and 1995 by age group

| Age group | Urban women | | | | Rural women | | | |
|---|---|---|---|---|---|---|---|---|
| | Mastec-tomy % | BCT only % | Axillary surgery % | No. of women *n* | Mastec-tomy % | BCT only % | Axillary surgery % | No. of women *n* |
| **1992** | | | | | | | | |
| <40 | 60 | 40 | 87 | 125 | 75 | 25 | 91 | 32 |
| 40–49 | 55 | 45 | 82 | 371 | 68 | 32 | 87 | 108 |
| 50–59 | 62 | 38 | 83 | 349 | 66 | 34 | 82 | 91 |
| 60–69 | 63 | 37 | 81 | 320 | 65 | 35 | 75 | 124 |
| 70–79 | 61 | 39 | 70 | 264 | 73 | 27 | 78 | 95 |
| 80+ | 46 | 54 | 46 | 111 | 57 | 43 | 57 | 30 |
| All ages | 59 | 41 | 78 | 1540 | 67 | 33 | 80 | 480 |
| **1995** | | | | | | | | |
| <40 | 56 | 44 | 89 | 165 | 66 | 34 | 97 | 38 |
| 40–49 | 57 | 43 | 89 | 461 | 56 | 44 | 92 | 130 |
| 50–59 | 52 | 48 | 87 | 547 | 61 | 39 | 85 | 192 |
| 60–69 | 51 | 49 | 81 | 479 | 59 | 41 | 82 | 164 |
| 70–79 | 54 | 46 | 73 | 369 | 69 | 31 | 80 | 142 |
| 80+ | 49 | 51 | 52 | 128 | 56 | 44 | 44 | 68 |
| All ages | 53 | 47 | 82 | 2149 | 61 | 39 | 81 | 734 |

*© 2001 Cancer Research Campaign* 

**Table 2** ORs for mastectomy and axillary surgery by age in women with invasive breast cancer in 1992 and 1995

| Age | 1992 OR[a] | 1992 95% CI | | 1995 OR[a] | 1995 95% CI | |
|---|---|---|---|---|---|---|
| Mastectomy | | | | | | |
| 24–39 | 0.99 | 0.68 | 1.45 | 1.13 | 0.82 | 1.55 |
| 40–49 | 0.80 | 0.61 | 1.03 | 1.09 | 0.87 | 1.35 |
| 50–59 | 1.00 | | | 1.00 | | |
| 60–69 | 0.98 | 0.75 | 1.30 | 0.92 | 0.75 | 1.14 |
| 70–79 | 1.02 | 0.76 | 1.37 | 1.16 | 0.92 | 1.46 |
| 80+ | 0.55 | 0.37 | 0.81 | 0.85 | 0.62 | 1.17 |
| | | *P*-value | 0.02 | | *P*-value | 0.3 |
| Axillary surgery | | | | | | |
| 24–39 | 1.48 | 0.86 | 2.55 | 1.52 | 0.90 | 2.55 |
| 40–49 | 1.02 | 0.72 | 1.44 | 1.35 | 0.96 | 1.90 |
| 50–59 | 1.00 | | | 1.00 | | |
| 60–69 | 0.79 | 0.56 | 1.11 | 0.67 | 0.50 | 0.89 |
| 70–79 | 0.52 | 0.37 | 0.73 | 0.46 | 0.35 | 0.62 |
| 80+ | 0.19 | 0.13 | 0.29 | 0.15 | 0.11 | 0.22 |
| | | *P*-value | <0.001 | | *P*-value | <0.001 |

[a]ORs were adjusted for urban or rural residence, SES and COB.

**Table 3** Size of breast cancer and type of surgical breast procedure by urban and rural place of residence in women 40–69 years of age in 1992 and 1995

| | 1992 | | | | | 1995 | | | | |
|---|---|---|---|---|---|---|---|---|---|---|
| | Urban | | Rural | | | Urban | | Rural | | |
| | Mastectomy | BCT only | Mastectomy | BCT only | All women | Mastectomy | BCT only | Mastectomy | BCT only | All women |
| | *n* = 898 | | *n* = 271 | | | *n* = 659 | | *n* = 200 | | |
| | % | % | % | % | | % | % | % | % | |
| Cancer size | | | | | | | | | | |
| 0–0.9 cm | 50 | 50 | 70 | 30 | 128 | 36 | 64 | 53 | 47 | 160 |
| 1–1.9 cm | 49 | 51 | 55 | 45 | 455 | 50 | 50 | 53 | 47 | 386 |
| 2.0–2.9 cm | 62 | 38 | 63 | 37 | 329 | 64 | 36 | 50 | 50 | 206 |
| 3.0+ cm | 81 | 19 | 87 | 13 | 257 | 79 | 21 | 87 | 13 | 107 |
| All sizes | 60 | 40 | 65 | 35 | 1169 | 54 | 46 | 58 | 43 | 859 |

Lobular cancer, although significantly associated with mastectomy after adjustment for size and spread of cancer at diagnosis in 1992 (OR 1.7. 95% CI 1.1–2.7), was less strongly associated with it in 1995 (OR 1.3, 95% CI 0.8–2.1; Table 4).

The odds of mastectomy did not vary significantly with socio-economic status after adjustment for age and size and spread of cancer at diagnosis (Table 4). Under similar conditions, mastectomy was, if anything, most prevalent in hospitals in which moderate numbers of breast procedures were done rather than in those with few or many.

### Axillary surgery

There were 1 580 women who had axillary surgery recorded in the linked dataset in 1992 (78%) and 2,354 (82%) in 1995. Axillary surgery was more often done in association with mastectomy (91% in 1992, 93% in 1995) than with BCT (58% in 1992, 67% in 1995) and increased between 1992 and 1995, mainly with BCT.

Urban women were more likely to have axillary surgery with BCT than rural women in 1992 and 1995 (Table 1) while the odds of axillary surgery fell nearly 10-fold with increasing age (*P*-value for age <0.001 in each year; Table 2).

In comparison with cancers <1.0 cm in diameter, the odds of axillary surgery in 1992 was significantly higher only for breast cancers larger than 2 cm (OR for 2–2.9 cm = 1.8 95% CI 1.0–3.3; OR for 3+ cm = 1.6 95% CI 0.9–3.1) in women 40–69 years of age (Table 5). In 1995, it was some two-fold or more higher for all cancers 1 cm or more in diameter in comparison with those <1.0 cm; the highest odds ratio was in 2.0–2.9 cm cancers (OR = 3.3 95% CI 1.7–6.7).

Regional spread of cancer at diagnosis was a strong predictor of axillary surgery in 1992 (OR 2.4, 95% CI 1.6–3.7, with reference to localized cancer) but not in 1995 (OR 1.5, 95% CI 0.9–2.6).

Axillary surgery was much more likely in hospitals with moderate or high numbers of breast procedures in 1992 than in those with low numbers (Table 5). This difference was not present in 1995.

### DISCUSSION

Breast conserving surgery increased in NSW between 1992 and 1995, mainly in women with small, localized breast cancers. For larger breast cancers or cancers that had spread beyond the breast, mastectomy remained the most common surgical treatment. By 1995, most women had axillary surgery regardless of cancer size or reported regional spread of the cancer. Women

*© 2001 Cancer Research Campaign*

**Table 4** Association of mastectomy with age, SES, urban or rural residence cancer spread, histopathological type, cancer size, and the hospital volume of surgical procedures in women 40–69 years of age diagnosed with invasive breast cancer in 1992 and 1995

| | **1992 (*n* = 1085)** | | | **1995 (*n* = 799)** | | |
|---|---|---|---|---|---|---|
| | **OR (adjusted)** | **95%CI** | | **OR (adjusted)** | **95% CI** | |
| Age (years) | | | | | | |
| 40–49 | 0.7 | 0.5 | 0.9 | 1.1 | 0.8 | 1.6 |
| 50–59 | 1.0 | | | 1.00 | | |
| 60–69 | 1.0 | 0.7 | 1.4 | 1.1 | 0.8 | 1.6 |
| | | *P*-value | 0.01 | | *P*-value | 0.7 |
| SES | | | | | | |
| 859–952 | 1.0 | | | 1.0 | | |
| 953–979 | 0.6 | 0.4 | 1.0 | 0.8 | 0.5 | 1.3 |
| 9810–1000 | 0.7 | 0.4 | 1.1 | 1.2 | 0.7 | 1.9 |
| 1001–1057 | 0.8 | 0.5 | 1.2 | 1.1 | 0.7 | 1.8 |
| 1058–1170 | 0.7 | 0.5 | 1.2 | 0.8 | 0.5 | 1.3 |
| | | *P*-value | 0.4 | | *P*-value | 0.5 |
| Residence | | | | | | |
| Urban | 1.0 | | | 1.0 | | |
| Rural | 1.3 | 1.0 | 1.9 | 1.3 | 0.9 | 1.9 |
| | | *P*-value | 0.09 | | *P*-value | 0.3 |
| Extent of cancer | | | | | | |
| Localized | 1.0 | | | 1.0 | | |
| Regional | 2.0 | 1.5 | 2.7 | 2.0 | 1.4 | 2.7 |
| Metastatic | 0.8 | 0.3 | 2.3 | 1.1 | 0.2 | 6.8 |
| | | *P*-value | <0.001 | | *P*-value | <0.001 |
| Histopathological type | | | | | | |
| Ductal | 1.0 | | | 1.0 | | |
| Lobular | 1.7 | 1.1 | 2.7 | 1.3 | 0.8 | 2.1 |
| Special types | 0.7 | 0.4 | 1.1 | 0.5 | 0.3 | 1.0 |
| | | *P*-value | 0.01 | | *P*-value | 0.07 |
| Size (cm) | | | | | | |
| 0–0.9 | 1.0 | | | 1.0 | | |
| 1.0–1.9 | 0.7 | 0.5 | 1.1 | 1.4 | 1.0 | 2.1 |
| 2.0–2.9 | 1.1 | 0.7 | 1.7 | 1.8 | 1.1 | 2.9 |
| 3+ | 3.1 | 1.8 | 5.3 | 5.6 | 2.9 | 10.7 |
| | | *P*-value | <0.001 | | *P*-value | <0.001 |
| Hospital volume | | | | | | |
| 1–10 procedures | 1.0 | | | 1.0 | | |
| 11–20 procedures | 1.5 | 0.9 | 2.6 | 1.3 | 0.6 | 2.5 |
| 21+ procedures | 0.9 | 0.6 | 1.4 | 1.3 | 0.7 | 2.1 |
| | | *P*-value | 0.04 | | *P*-value | 0.7 |

with the smallest (<1 cm) cancers, however, were less likely to have nodes removed. Axillary surgery fell steeply with increasing age from 25–39 years to the oldest women 80+ years. Our study depended on the linkage of two sets of routinely collected, computerised health records, a well-regarded method of enriching individually collected datasets for epidemiological and health services research.

BCT rates also increased in the USA and UK in the late 1980s and early 1990s (Chouillet et al, 1994; Richards et al, 1996; Nattinger et al, 1996; Lazovich et al, 1997) mainly, as in NSW, in women with smaller cancers (Lazovich et al, 1997). In the USA, rates of stage I cancers, which doubled from 1983 to 1996, were already at a high level by 1990 (National Cancer Institute, 1999) while BCT rates rose steadily from 1990 to 1995 (Lazovich et al, 1997; Nattinger et al, 2000). Thus, increasing BCT rates in the 1990s appear not to be due solely to increasing rates of smaller cancers, though BCT rates in the USA were higher in women with smaller cancers (Lazovich et al, 1997), as in NSW.

BCT use appears to have peaked in the UK and Netherlands in the late 1980s to 1990 at around 40% of women with breast cancer (de Koning et al, 1994; Haward et al, 1999). The high rate in 1995 in NSW (45%) may prove to have been a peak and, if so, would be consistent with De Koning et al's (1994) predicted limit for BCT at around 42% of all newly diagnosed breast cancers (de Koning et al, 1994).

Mastectomy rates were higher in non-metropolitan than urban or metropolitan women, as in the USA (Howe et al, 1992; Nattinger et al, 1996; Lazovich et al, 1997), independently of any regional differences in breast cancer size and stage at diagnosis (Table 4). These higher rates were unlikely to be due to poorer health and a consequent possible lower tolerance of adjuvant therapy since rural Australian women, if anything, are healthier than urban women (Mathers, 1994; Australian Institute of Health and Welfare, 1998). Australian rural residents generally have a low use of medical services and less access to specialist medical care (Australian Institute of Health and Welfare, 1998) and their higher mastectomy

*© 2001 Cancer Research Campaign*

**Table 5** Association of axillary surgery with age, SES, cancer spread, cancer size, and the hospital volume of surgical procedures in women 40–69 years of age diagnosed with invasive breast cancer in 1992 and 1995

| | **1992 (*n* = 1088)** | | | **1995 (*n* = 794)** | | |
|---|---|---|---|---|---|---|
| | **OR (adjusted)** | **95% CI** | | **OR (adjusted)** | **95% CI** | |
| Age (years) | | | | | | |
| 40–49 | 1.0 | 0.7 | 1.6 | 0.9 | 0.5 | 1.7 |
| 50–59 | 1.0 | | | 1.0 | | |
| 60–69 | 0.9 | 0.6 | 1.4 | 0.5 | 0.3 | 0.9 |
| | | *P*-value | 0.8 | | *P*-value | 0.04 |
| SES | | | | | | |
| 859–952 | 1.0 | | | 1.0 | | |
| 953–979 | 0.9 | 0.4 | 1.7 | 0.6 | 0.3 | 1.2 |
| 980–1000 | 0.6 | 0.3 | 1.1 | 1.2 | 0.5 | 2.8 |
| 1001–1057 | 1.3 | 0.6 | 2.6 | 0.6 | 0.3 | 1.3 |
| 1058–1170 | 0.5 | 0.3 | 1.0 | 0.9 | 0.4 | 1.8 |
| | | *P*-value | 0.008 | | *P*-value | 0.2 |
| Extent of cancer | | | | | | |
| Localized | 1.0 | | | 1.00 | | |
| Regional | 2.4 | 1.6 | 3.7 | 1.5 | 0.9 | 2.6 |
| Metastatic | 0.7 | 0.2 | 2.3 | – | – | – |
| | | *P*-value | <0.001 | | *P*-value | 0.1 |
| Size | | | | | | |
| 0–0.9 cm | 1.0 | | | 1.0 | | |
| 1.0–1.9 cm | 1.0 | 0.6 | 1.8 | 2.2 | 1.3 | 3.8 |
| 2.0–2.9 cm | 1.8 | 1.0 | 3.3 | 3.3 | 1.7 | 6.7 |
| 3+ cm | 1.6 | 0.9 | 3.1 | 1.8 | 0.8 | 3.9 |
| | | *P*-value | 0.04 | | *P*-value | 0.003 |
| Hospital volume | | | | | | |
| 1–10 procedures | 1.0 | | | 1.0 | | |
| 11–20 procedures | 2.0 | 1.1 | 3.6 | 1.1 | 0.4 | 3.1 |
| 21+ procedures | 2.6 | 1.6 | 4.1 | 1.1 | 0.5 | 2.5 |
| | | *P*-value | <0.001 | | *P*-value | 1.0 |

rates may have been due to less specialized care (Howe et al, 1992) and less access to radiotherapy, a recommended accompaniment to BCT (National Institute of Health 1990).

Although we did not find that hospital caseload was a strong predictor of BCT, higher surgeon caseload had been in a 1995 national survey (Hill et al, 1999) and low surgeon caseload a predictor of mastectomy in urban NSW in 1992 (Taylor et al, 1999). Perhaps, as in the USA, use had become more uniform across hospital settings by the 1990s (Lazovich et al, 1997). We found little evidence that SES influenced BCT rates.

Axillary surgery increased slightly from 1992 to a high percentage of NSW women in 1995. The patterns of axillary surgery mirrored US experience, accompanying mastectomy more often than BCT and being inversely related to women's ages and only weakly related to cancer size (Du et al, 1999; Nattinger et al, 2000). Since larger cancer size predicts positive nodes and young women have a higher probability of positive nodes than women of other ages (Olivotto et al, 1998; Yiangou et al, 1999), axillary surgery appeared to be appropriately targeted to younger women but less evidently to larger breast cancers.

If 'appropriate' (arguably optimal) surgery is defined as 'according to recommendation', then just over 80% of women in this study had optimal surgical treatment of the axilla. Axillary dissection in early breast cancer aims to control disease by reducing recurrence and to provide stage information for decisions about systemic therapy. In addition, women place a high value on knowing the status of the lymph nodes even when unfavourable, despite poor outcomes in about 40% of women including arm dysfunction such as pain and loss of grip strength (Galper et al, 2000). Attention, appropriately, to women's preferences may account for some of the 20% of women who appear not to have optimal treatment of the axilla.

Women also have personal reasons for choosing non-conservative surgery. Surgeons reported that 25% of Australian women with early breast cancer in 1995 chose non-conservative surgery for reasons such as concerns about recurrence or treatment by radiotherapy, which were sometimes age- or residence-related (Hill et al, 1999). This preference, together with those in relation to axillary surgery, suggests that benchmarks should be set not only for the proportions of women having particular procedures but also for the proportions who were informed about the options and their pros and cons, and whether their preferences were honoured.

Our results show that detailed and informative analyses of patterns of breast cancer care in a whole population can be gained through linkage of routinely collected cancer registry and inpatient statistics records when the cancer registry does not routinely collect treatment data. This may be a cost-effective alternative to separate collection of treatment data by cancer registries.

### ACKNOWLEDGEMENTS

Data analyses were set up by Kevin McGeechan and continued by Jane Ambrose. The NSW Central Cancer Registry, which is managed and operated by the NSW Cancer Council under contract

*© 2001 Cancer Research Campaign*

BJOC 01-1932 668-673 20/8/01 3:10 pm Page 673

to the NSW Health Department, supplied the data on invasive breast cancer in 1992 and 1995. The NSW Health Department made available a linked file of surgical procedures recorded in the NSW Inpatient Statistics Collection and women with breast cancer registered by the Cancer Registry in 1992 and 1995. The National Breast Cancer Centre is funded by the Commonwealth Government of Australia.

### REFERENCES

Australian Institute of Health and Welfare (1998) *Health in rural and remote Australia*. Cat.No.PHE 6. Australian Institute of Health and Welfare: Canberra

Baldwin JA (1982) The Oxford Record linkage study as a medical information system. *Proc R Soc Med* **65**: 237–239

Chouillet AM, Bell CM and Hiscox JG (1994) Management of breast cancer in southeast England. *BMJ* **308**: 168–171

de Koning HJ, van Dongen JA and van der Maas PJ (1994) Changes in use of breast-conserving therapy in years 1978–2000. *Br J Cancer* **70**: 1165–1170

Du X, Freeman JL and Goodwin JS (1999) The declining use of axillary dissection in patients with early stage breast cancer. *Breast Cancer Res Treat* **53**: 137–144

Galper SR, Lee SJ, Tao ML, Troyan S, Kaelin CM, Harris JR and Weeks JC (2000) Patient preferences for axillary dissection in the management of early-stage breast cancer. *J Natl Cancer Inst* **92**: 1681–1687

Haward R, Johnston C and Sainsbury R (1999) Achieving consistency in breast cancer care: An analytical tool to measure change. *Cancer Strategy* **1**: 65–72

Hill D, Jamrozik K, White V, Collins J, Boyages J, Shugg D, Pruden M, Giles G and Byrne M (1999) *Surgical management of breast cancer in Australia in 1995*. NHMRC National Breast Cancer Centre

Hobbs MST and McCall MG (1970) Health statistics and record linkage in Australia. *J Chronic Dis* **23**: 375–381

Howe HL, Katterhagen JG, Yates J and Lehnherr M (1992) Urban-rural differences in the management of breast cancer. *Cancer Causes Control* **3**: 533–539

Kahn LH, Blustein J, Arons RR, Yee R and Shea S (1996) The validity of hospital administrative data in monitoring variations in breast cancer surgery. *Am J Public Health* **86**: 243–245

Kricker A, Farac K, Smith D, Sweeny A, McCredie M and Armstrong BK (1999) Breast cancer in New South Wales in 1972–1995: tumor size and the impact of mammographic screening. *Int J Cancer* **81**: 877–880

Lazovich D, White E, Thomas DB, Moe RE and Taplin S (1997) Change in the use of breast-conserving surgery in western Washington after the 1990 NIH Consensus Development Conference. *Arch Surg* **132**: 418–423

Mathers C (1994) *Health differentials among older Australians aged 25–64 years*. Australian Institute of Health and Welfare. Health Monitoring Series No. 1: Canberra

McGeechan K, Kricker A, Armstrong B and Stubbs J (1998) Evaluation of linked cancer registry and hospital records of breast cancer. *Aust N Z J Public Health* **22**: 765–770

National Cancer Institute (1999) *Surveillance, Epidemiology and End Results (SEER) Cancer Incidence Public-Use Database, 1973–1996*

National Health & Medical Research Council (1995) *NHMRC Clinical practice guidelines for the management of early breast cancer*. NHMRC: Canberra

National Institute of Health (1990) Treatment of Early-Stage Breast Cancer. *NIH Consensus Statement Online* 1990 Jun 18–21; **8**(6): 1–19

Nattinger AB, Hoffman RG, Shapiro R, Gottlieb MS and Goodwin JS (1996) The effect of legislative requirements on the use of breast-conserving surgery. *N Engl J Med* **335**: 1035–1040

Nattinger AB, Hoffmann RG, Kneusel RT and Schapira MM (2000) Relation between appropriateness of primary therapy for early-stage breast carcinoma and increased use of breast-conserving surgery. *Lancet* **356**: 1148–1153

Olivotto IA, Jackson JS, Mates D, Andersen S, Davidson W, Bryce CJ and Ragaz J (1998) Prediction of axillary lymph node involvement of women with invasive breast carcinoma: a multivariate analysis. *Cancer* **83**: 948–955

Richards MA, Wolfe CD, Tilling K, Barton J, Bourne HM and Gregory WM (1996) Variations in the management and survival of women under 50 years with breast cancer in the South East Thames region. *Br J Cancer* **73**: 751–757

Taylor R, Stubbs JM, Langlands AO and Boyages J (1999) Predictors of mastectomy for women with breast cancer in the greater western region of Sydney. *The Breast* **5**: 116–121

Yiangou C, Shousha S and Sinnett HD (1999) Primary tumour characteristics and axillary lymph node status in breast cancer. *Br J Cancer* **80**: 1974–1978

© 2001 Cancer Research Campaign
